# The central nervous system tumor methylation classifier changes neuro-oncology practice for challenging brain tumor diagnoses and directly impacts patient care

**DOI:** 10.1186/s13148-019-0766-2

**Published:** 2019-12-05

**Authors:** Shirin Karimi, Jeffrey A. Zuccato, Yasin Mamatjan, Sheila Mansouri, Suganth Suppiah, Farshad Nassiri, Phedias Diamandis, David G. Munoz, Kenneth D. Aldape, Gelareh Zadeh

**Affiliations:** 1 Division of Neurosurgery, University Health Network, University of Toronto, Toronto, Ontario Canada; 2 MacFeeters-Hamilton Center for Neuro-Oncology, Princess Margaret Cancer Center, Princess Margaret Cancer Research Tower, 101 College Street, 14th floor, Room 601, Toronto, ON M5G 1L7 Canada; 3 Laboratory Medicine Program, University Health Network, Toronto, Ontario Canada; 4 Department of Laboratory Medicine, St. Michael’s Hospital, Toronto, Ontario Canada; 5 Laboratory of Pathology, Center for Cancer Research, National Cancer Institute, Building 10, Room 2S235, Bethesda, MD 20892-1500 USA

**Keywords:** Central nervous system, Neuro-oncology, Epigenetics, Translational research

## Abstract

**Background:**

Molecular signatures are being increasingly incorporated into cancer classification systems. DNA methylation-based central nervous system (CNS) tumor classification is being recognized as having the potential to aid in cases of difficult histopathological diagnoses. Here, we present our institutional clinical experience in integrating a DNA-methylation-based classifier into clinical practice and report its impact on CNS tumor patient diagnosis and treatment.

**Methods:**

Prospective case review was undertaken at CNS tumor board discussions over a 3-year period and 55 tumors with a diagnosis that was not certain to two senior neuropathologists were recommended for methylation profiling based on diagnostic needs. Tumor classification, calibrated scores, and copy number variant (CNV) plots were obtained for all 55 cases. These results were integrated with histopathological findings to reach a final diagnosis. We retrospectively reviewed each patient's clinical course to determine final neuro-pathology diagnoses and the impact of methylation profiling on their clinical management, with a focus on changes that were made to treatment decisions.

**Results:**

Following methylation profiling, 46 of the 55 (84%) challenging cases received a clinically relevant diagnostic alteration, with two-thirds having a change in the histopathological diagnosis and the other one-third obtaining clinically important molecular diagnostic or subtyping alterations. WHO grading changed by 27% with two-thirds receiving a higher grade. Patient care was directly changed in 15% of all cases with major changes in clinical decision-making being made for these patients to avoid unnecessary or insufficient treatment.

**Conclusions:**

The integration of methylation-based CNS tumor classification into diagnostics has a substantial clinical benefit for patients with challenging CNS tumors while also avoiding unnecessary health care costs. The clinical impact shown here may prompt the expanded use of DNA methylation profiling for CNS tumor diagnostics within prominent neuro-oncology centers globally.

## Introduction

The classification and diagnosis of central nervous system (CNS) tumors, as with all neoplastic processes, is essential in determining optimal patient treatment decisions. Histopathological features have traditionally been used to classify tumors using World Health Organization (WHO) criteria [1]. However, in the past decade, the value of integrating molecular alterations into WHO classification criteria has been widely recognized [2–5]. Specific molecular features that have been incorporated into the 2016 WHO classifications include IDH mutation and 1p/19q co-deletion statuses for gliomas as well as definitions of subtypes for medulloblastomas and ependymomas [1, 6].

Genome-wide DNA methylation differences have been used to determine the cell of origin and classify many non-CNS tumors [7–10]. Additionally, a recent seminal publication has demonstrated its utility in classifying CNS tumors with improved diagnostic accuracy in comparison to the standard of care histopathological analysis and single molecular tests [11]. This CNS tumor classifier was built based on methylation profiling of 2682 brain tumor cases across 82 different tumor subtypes and then it was applied to 1104 additional cases to determine diagnostic utility. Of these 1104 cases in their cohort, 12% received a new integrated diagnosis when methylation profiling results were added to standard of care histopathological testing. To date, however, the impact of methylation classifier on clinical outcome has not been well established.

In this study, we describe a single institutional experience of implementing the CNS tumor methylation-based classifier into clinical practice. Through key case descriptions, we illustrate the impact on patient outcomes after integrating methylation profiling results in their care. A substantial proportion of patients (84%) had important treatment decision changes made after methylation profiling that modified their CNS tumor diagnosis. A significant percentage (15%) had a resulting change in the clinical treatment course. The value of rapidly adopting and integrating the methylation-based CNS tumor classifier into clinical practice is highlighted in this work.

## Materials and methods

### Patient cohort selection

Between November 1, 2015 and September 30, 2018, all brain tumor cases reviewed at UHN multidisciplinary CNS tumor board meetings and selected for methylation analysis were included in this study. Reasons to select cases included (1) challenging diagnoses including those with unusual morphological features where two independent experienced neuropathologists were uncertain of the final diagnosis based on histopathological assessment and existing molecular markers, (2) diffuse gliomas with immunohistochemistry (IHC) antibody-negative mutant-IDH1 (R132H) status to determine final IDH status, (3) ependymomas or medulloblastomas requiring molecular subtyping, (4) indeterminant fluorescence in situ hybridization (FISH) results for 1p/19q co-deletion status in diffuse gliomas needing validation, and (5) discrepancies between clinical or imaging features and histopathological diagnoses that raised concern for alternative diagnoses. All standard of care FISH, IHC, and other molecular testing were completed prior to methylation profiling consideration as is routine practice at our institution.

### DNA methylation analysis

Tumor samples from formalin-fixed paraffin-embedded tissue specimens including five scrolls or at least ten unstained slides with 5–10 μm in thickness were utilized and areas with the available tumor cellularity over 70% were preferred. Extracted DNA (150–500 ng) was bisulfite-converted (EZ DNA Methylation Kit, Zymo Research D5001). Samples were processed in our institutional genomics facility that combined samples from multiple sources to process in batches of 12 for the Illumina Infinium HumanMethylation450 BeadChip (450k) array or 8 for the MethylationEPIC BeadChip (850k) array. Standard quality controls confirmed adequate tumor purity/quality, bisulfite conversion, and DNA quality. IDAT files were uploaded to either version 11b2 or 11b4 of the online CNS tumor methylation classifier (https://www.molecularneuropathology.org) and reports were produced as shown by Capper et al. [11].

### Diagnostic integration

The methylation results from the classifier include the methylation classification, calibrated scores reflecting prediction scores of methylation class diagnoses, and chromosomal copy number variation (CNV) plots generated from raw methylation data. Calibrated scores were assessed based on the German Cancer Research Center protocol with a preference for calibrated scores ≥ 0.9 but inclusion between 0.3 and 0.9 in the context of low tumor-cell content [12]. Deletions, amplifications, and losses or gains were assessed visually in the CNV plots for specific diagnostic alterations (e.g., 1p/19q whole arm loss for oligodendroglioma) using the approach described by the German Cancer Research Center group [13]. MGMT methylation was assessed by the classifier using a methylation probability cutoff of 0.3582 [12, 14]. The diagnostic workflow included a re-evaluation of original histopathology as well as all standard of care molecular testing results, integration of these results with methylation classification and CNV plots to determine the final diagnosis, and feedback of any diagnostic changes to treating clinicians in tumor board discussions.

### Clinical data collection

Data that were collected both prospectively and retrospectively were needed to determine the clinical course and oncological outcomes. Fields collected included demographics, preoperative clinical factors, neuroimaging findings, surgical details, pathology results, patient treatment decisions made prior to and following completion of methylation profiling, treatment complications, and follow-up clinical status.

## Results

### Cohort characteristics

Between November 1, 2015, and September 30, 2018, a total of 1670 operative cases were reviewed at UHN CNS tumor board discussions. Of all cases, 66 represented diagnostic challenges and thereby fit the criteria for methylation profiling. A total of 55 cases were included in this study with 11 cases excluded due to insufficient tumor quantity/quality or no classifier tumor match. The median turnaround time for methylation profiling was 25 days. Clinical characteristics of the cohort along with reasons to select patients for methylation profiling are shown in Table 1. The average age was 41 years (range = 18–71) with equal distribution across sexes and the majority having supratentorial tumors. Three-quarters of patients remain alive and average follow-up is over 2 years. The key reason for identifying a case as challenging included an uncertain diagnosis by two expert neuropathologists with differential pathological diagnoses being given in 20/55 (36%) cases and unusual morphological features being present for the proposed diagnosis in 13/55 (24%). The distribution of cases across WHO tumor types reflects a general CNS oncology practice and is shown in Table 2, with the majority being diffuse gliomas.

**Table 1:** Cohort characteristics

Characteristic			
		Mean	Range
Age (years)^a^		41.0	18–71
Follow-up since surgery (years)^b^		2.1	0.1–15.5
		*N*	%
Gender^a^	Male	25	49
Tumor location^b^	Supratentorial	38	81
	Infratentorial, intracranial	7	15
	Spinal, intradural	2	4
Surgery for recurrent tumor^b^		8	17
Extent of resection^d^	Subtotal resection	30	68
Current status^b^	Well/stable disease	35	74
	Palliative/deceased	12	26
Indication for methylation profiling^e^	Challenging diagnosis with differential given	20	36
	Diagnoses without classical features	13	24
	Final determination of IDH status	11	20
	Molecular subtyping	4	7
	Final determination of 1p/19q co-deletion status	4	7
	Clinical discrepancy with pathologic diagnosis	3	5
Initial WHO grade^e^	I	8	15
	II	15	27
	III	13	24
	IV	15	27
	Not graded	4	7

^a^*n* = 51

^b^*n* = 47

^c^*n* = 45

^d^*n* = 44

^e^*n* = 55

**Table 2:** Initial histopathological diagnoses

Tumor type (*n* = 55)		*N*	%
Diffuse glioma	Diffuse astrocytoma, IDH mutant	4	7
	Diffuse astrocytoma, IDH1 (R132H) negative	2	4
	Anaplastic astrocytoma, IDH mutant	2	4
	Anaplastic astrocytoma, IDH1 (R132H) negative	3	5
	Oligodendroglioma, IDH1 (R132H) negative, 1p/19q co-deleted	2	4
	Anaplastic oligodendroglioma, IDH1 (R132H) negative, 1p/19q co-deleted	3	5
	Glioblastoma, IDH1 (R132H) negative	11	20
	Glioma NOS	3	5
	Low grade glioma	1	2
	Diffuse low grade glioma	1	2
Other astrocytic	Pilocytic astrocytoma	1	2
	PXA	1	2
	Anaplastic PXA	2	4
Ependymal	Subependymoma	2	4
	Ependymoma	1	2
	Anaplastic ependymoma	3	5
Choroid plexus	Atypical choroid plexus papilloma	1	2
Neuronal	Dysembryoplastic neuroepithelial tumor	1	2
	Ganglioglioma variant	2	4
Embryonal	Medulloblastoma	3	5
Nerves	Schwannoma	1	2
Meningioma	Atypical meningioma	1	2
Mesenchymal	SFT/hemangiopericytoma	1	2
Metastatic	Melanocytic lesion NOS	1	2
	Metastatic high-grade NE neoplasm	1	2
Non-neoplastic	Meningioangiomatosis	1	2

*NOS* not otherwise specified, *PXA* pleomorphic xanthoastrocytoma, *SFT* solitary fibrous tumor, *NE* neuroendocrine

### Diagnostic impact of methylation profiling

**Fig. 1:**
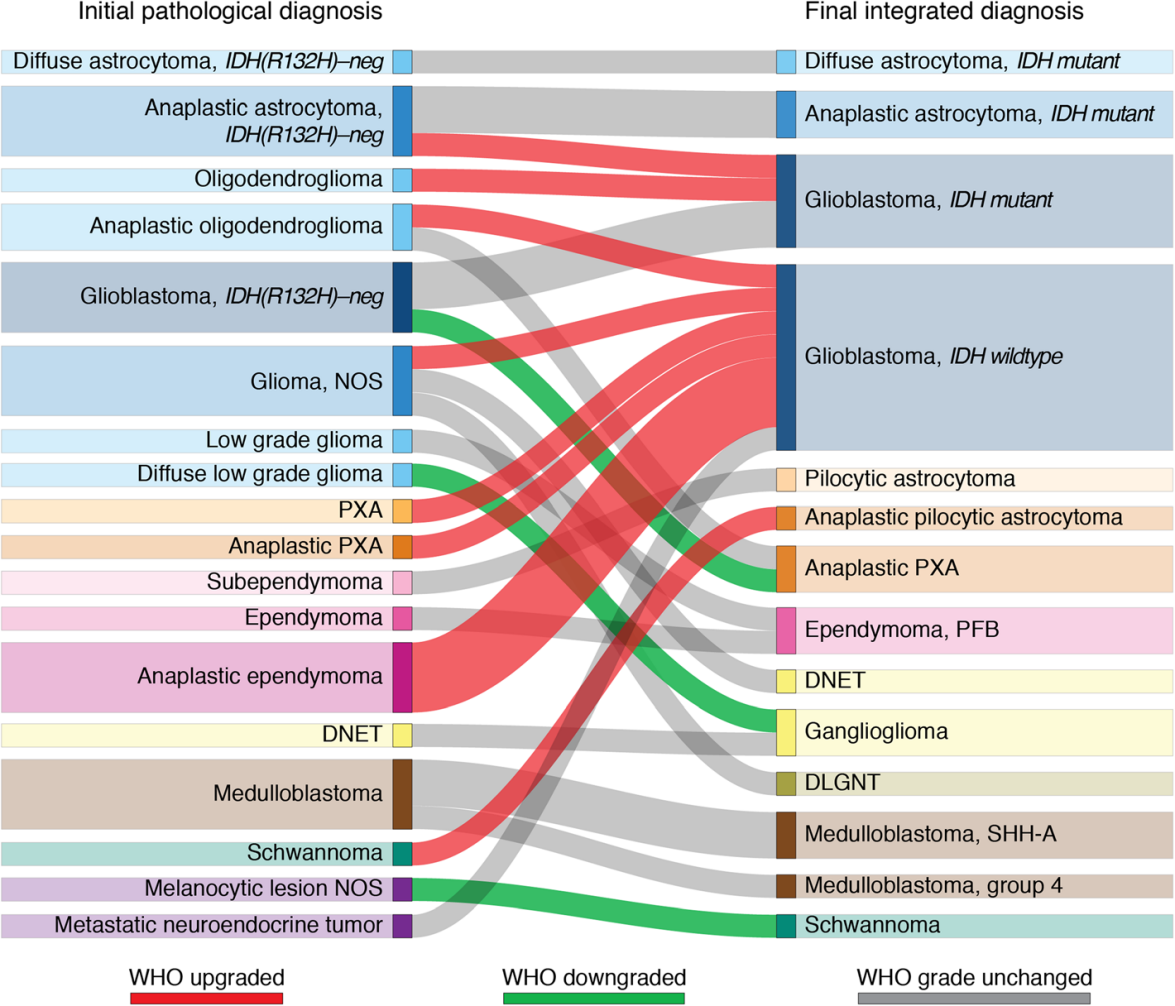
Establishing new diagnoses based on methylation profiling of CNS tumors NOS, not otherwise specified; PXA, pleomorphic xanthoastrocytoma; DNET, dysembryoplastic neuroepithelial tumor; DLGNT, diffuse leptomeningeal glioneuronal tumor Methylation profiling led to a change from initial histopathological diagnosis (left) to the final integrated diagnosis (right) or to the identification of a new clinically relevant molecular subtype in 29 cases. This subset includes patients with a newly identified diagnosis [13], a resolved differential diagnosis different from the top initial diagnosis [7], or a new clinically relevant molecular subtype [9]. WHO grading changes are shown in red (upgrading) and green (downgrading).

Table 3 shows that a diagnostic alteration made between initial pathological diagnosis and final integrated molecular diagnoses using methylation profiling was observed in 84% of cases (46/55). These cases include 24% (13/55) with a change in the diagnostic entity, 31% (17/55) with a resolved differential diagnosis, and 29% (16/55) having clinically relevant molecular subtyping determinations. The classifier changed or finalized the WHO grade in 27% (15/55), two-thirds of which were upgraded to a higher grade. Figure 1 summarizes the diagnostic impact in those cases with definitive changes in top diagnoses.

**Table 3:** Diagnostic impacts of methylation profiling

Category		*N*	%
Diagnostic effect^e^	Change in diagnosis	13	24
	Resolution of differential diagnosis	17	31
	Molecular refinement	16	29
	Diagnostic validation	9	16
WHO grade change^e^	Upgrade	10	18
	Downgrade	3	5
	Assign grade	2	4
	Unchanged	40	73
All molecular refinements^e^	Change in IDH mutation status	9	16
	Final determination of an unclear IDH mutation status	23	42
	Identification of molecular subtype	20	36
	Determination of 1p/19q co-deletion status	6	11

^b^*n* = 47

^e^*n* = 55

Evidence of an IDH mutation was identified in 9 of 24 diffuse glioma cases for which the R132H mutant-specific antibody was negative (6/9 showed ATRX loss by IHC and 3/9 were 1p/19q co-deleted by FISH), presumably reflecting non-canonical IDH mutations. The IDH1 mutation status in six cases determined by positive IHC staining for IDH1 (R132H)-specific antibody was confirmed by methylation analysis. CNV analyses also confirmed two of five 1p/19q co-deletions identified by FISH testing in oligodendrogliomas with the other three co-deletions by FISH being false positives. MGMT methylation status was identified by the classifier in all cases. Methylation profiling identified one MGMT methylated diffuse glioma with inconclusive MGMT pyrosequencing results and the results between both modalities were consistent in all of seven other diffuse gliomas that underwent pyrosequencing. Subtyping identified two SHH-A medulloblastomas and one from group 4 as well as two PFB ependymomas, one initially diagnosed as a low-grade glioma. The IDH wildtype glioblastoma cases were classified in the following subclasses: mesenchymal (10), RTK II (3), MYCN (1), and H3.3G34 (1) [3, 11].

### The impact of methylation profiling on clinical management

We defined a substantial clinical impact as a new diagnosis based on methylation profiling that would alter treatment decisions. Specific alterations include management decisions on surveillance imaging post-operatively as well as delivery of radiotherapy, chemotherapy, or targeted therapy. Table 4 summarizes the substantial number of patients (15%) whose treatment plan and adjuvant therapy were or would be changed after taking the new integrated diagnosis into consideration in management decisions. The median turnaround time for these specific cases was 32 days and results were made available prior to decision making regarding treatment. The seven cases are highlighted below:

**Table 4:** Clinical summary of cases with a significant methylation-mediated impact on patient care

Case no.	Initial pathology (*differential)	Initial treatment plan	Integrated diagnosis	New treatment plan	Methylation profiling impact
1	Glioma NOS *–IDH1 (R132H)-neg, high-grade features*	Offered FSRT/TMZ & adjuvant TMZ	DNET	Observation	1) Avoided unnecessary treatment 2) Resolved anxiety due to initial diagnosis/treatment
2	Anaplastic oligodendroglioma *–IDH1 (R132H)-neg, 1p/19q-codeletion*	1) Received FSRT 2) Planned for PCV	Anaplastic PXA	1) Hold PCV 2) Observe	Avoided unnecessary treatment
3	Glioma NOS *–IDH1 (R132H)-neg, anaplastic features*	Offered FSRT/TMZ and adjuvant TMZ	DLGNT	Observation	Avoided unnecessary treatment
4	1) GBM* 2) Anaplastic PXA *– IDH1 (R132H)-neg, BRAF (V600E)-mut, high-grade features*	FSRT/TMZ and adjuvant TMZ	Anaplastic PXA	Surgery, chemotherapy, and BRAF inhibitor after recurrence	1) Resolved depression due to unclear diagnosis 2) Avoided potential medical assisted death due to diagnosis given
5	Schwannoma *–SOX10-pos, S100-pos, Ki-67 10%*	FSRT	Anaplastic pilocytic astrocytoma *–Malignant glial tumor*	Provided full craniospinal radiation at recurrence	Received potentially insufficient initial treatment
6	1) PXA* 2) Other high-grade gliomas *– IDH1 (R132H)-neg, BRAF (V600E)-neg*	Considered reduced dosing for FSRT/TMZ & adjuvant TMZ	GBM, IDH wildtype	Treatment fully completed	Avoided possible insufficient initial treatment
7	Metastatic high-grade neuro-endocrine tumor *–TTF1-pos, IDH1 (R132H)-neg, well demarcated*	1) SRS post-op 2) FSRT and cisplatin/etopside for 1st recurrence 3) Surgery and WBRT for second recurrence	GBM, IDH wildtype	Temozolomide provided after second recurrence	1) Received insufficient treatment and recurred 2) May have avoided unnecessary invasive biopsies

*GBM* glioblastoma, *PXA* pleomorphic xanthoastrocytoma, *DLGNT* diffuse leptomeningeal glioneuronal tumor, *NOS* not otherwise specified, *DNET* dysembryoplastic neuroepithelial tumor, *SRS* single-dose stereotactic radiosurgery, *FSRT* fractionated stereotactic radiotherapy, *TMZ* temozolomide, *RT* radiotherapy

#### Case 1

A 20-year old female presented with seizures and imaging revealed a right frontal low-grade-infiltrative lesion consistent with a glioma. A gross total resection was achieved at surgery. The histopathological diagnosis was a glioma, not otherwise specified (NOS) without WHO grading which was IDH1 (R132H) antibody-negative, 1p/19q non-codeleted, and ATRX retained. Moderately severe nuclear atypia, areas of morphologically oligodendroglial-like differentiation, and focal microvascular proliferation raised the suspicion for a high-grade glioma including glioblastoma, although the tumor showed no evidence of necrosis and it was not mitotically active. After multidisciplinary tumor board consensus, the treatment offered was concurrent chemo-radiotherapy plus adjuvant temozolomide.

**Fig. 2:**
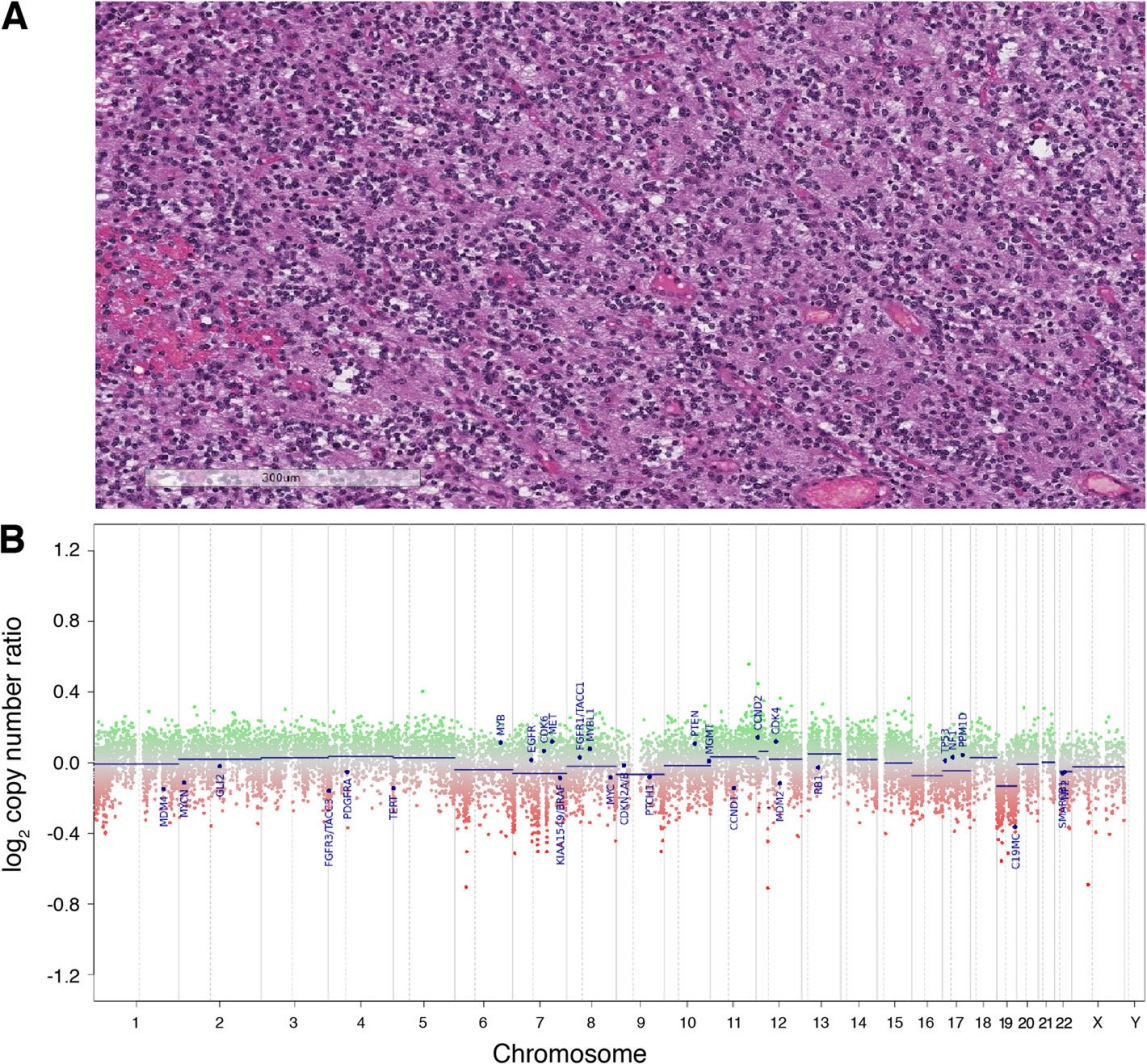
Histopathology and CNV plot for the tumor in Case 1 H&E stain and CNV plot for Case 1. a Hematoxylin and eosin stain of the tumor diagnosed as a Glioma, NOS with high-grade features and IDH1 (R132H) negative testing. b Relatively flat CNV plot supporting the integrated diagnosis of a dysembryoplastic neuro-epithelial tumor (DNET)

Methylation profiling was performed given the uncertainty in the diagnosis and grading of the tumor. The methylation classifier indicated that the tumor was a dysembryoplastic neuroepithelial tumor (DNET) with a calibrated score of 0.9, and this methylation class diagnosis was supported by a relatively flat CNV plot with no evidence of significant gene or chromosomal alterations. Two independent neuropathologists concurred with the new diagnosis upon review of the histopathology of the tumor, including the H&E stain and CNV plot shown in Fig. 2. Accordingly, the new management based on consensus of the tumor board discussion with the neuro-oncology team was observation and close surveillance imaging. This avoided cranial radiation for a young patient and she remains stable 13 months following initial diagnosis.

#### Case 2

A 23-year-old male presented with seizures and hemiplegia and imaging demonstrated a recurrent left temporal lesion. Following resection, the lesion was diagnosed histopathologically as a 1p/19q co-deleted anaplastic oligodendroglioma although it was noted that the IDH1 (R132H) antibody (IHC) was negative. With this diagnosis, the patient was treated with radiotherapy and was planned to receive PCV chemotherapy. Methylation profiling was pursued to confirm the diagnosis and determine the final IDH status. The tumor matched to the (anaplastic) pleomorphic xanthoastrocytoma (PXA) methylation class with a calibrated score of 0.94. The CNV plot identified a homozygous CDKN2A deletion and also confirmed that the proposed 1p/19q co-deletion by FISH testing was a false positive. Based on this diagnosis a significant change in treatment was made to hold PCV therapy and avoid the related side effect profile. The patient remains stable 14 months after surgery.

#### Case 3

A 25-year-old female presented with seizures and a recurrent right temporal tumor that was diagnosed as an oligodendroglioma eight years prior, with no specific molecular analysis performed. Surveillance imaging was carried out and at recurrence, the patient was referred to our institution for a second resection. The histopathology of the tumor resected in the second surgery showed a glioma, NOS that was not 1p/19q co-deleted by FISH testing and IDH1 (R132H) was negative by IHC. Based on the anaplastic features and absence of IDH1 (R132H) mutation, the management consensus after review in a tumor board discussion was concurrent chemoradiotherapy plus adjuvant temozolomide. Methylation profiling was initiated due to an unclear and challenging diagnosis. The tumor matched to the diffuse leptomeningeal glioneuronal tumor (DLGNT) methylation class with a calibrated score of 0.99. The CNV plot showed an isolated loss of chromosome 1. With the change in diagnosis, the new consensus on management after re-review at the neuro-oncology tumor board discussion was surveillance imaging and observation with no adjuvant treatment being given. The patient and imaging remain stable over 22 months.

#### Case 4

A 26-year-old female presented with seizures and a left temporal lesion called an astrocytoma, PXA, or other glioma based on MRI features. Following gross-total resection, the histopathological diagnosis was that of glioblastoma versus anaplastic PXA, and the tumor had high-grade morphological features, a BRAF V600E mutation, and was IDH1 (R132H) negative. Based on the uncertainty in differential diagnosis and concerning features on imaging for a high-grade lesion, the consensus on treatment was concurrent chemoradiotherapy plus adjuvant temozolomide. She suffered from considerable depression related to the possibility that she may have a glioblastoma and she considered medical assisted death due to this. Methylation profiling was pursued because of the unclear differential diagnosis and the deterioration in her clinical emotional status. The methylation class of the tumor was that of an (anaplastic) PXA with a calibrated score of 0.63. CNV plotting showed a homozygous CDKN2A loss and no other CNV findings consistent with glioblastoma. This classifier result and the calibrated score was interpreted as has been described by Capper et al. as supportive of the PXA diagnosis with additional confirmation from the BRAF mutation and homozygous CDKN2A loss [12]. The patient has since received redo-surgery, chemotherapy, and a BRAF inhibitor for recurrence. She is stable on the BRAF inhibitor after recurrence now 3 years since diagnosis and, notably, no longer pursuing medical assisted death.

#### Case 5

A 45-year-old male presented with sensorineural hearing loss and imaging revealed a left CP angle tumor considered a hemangiopericytoma versus schwannoma on MRI. After subtotal resection it was diagnosed as a SOX-10/S100 positive schwannoma with ancient change and rare mitotic activity along with a high Ki-67 of 10%. Accordingly, he received postoperative radiotherapy. He then progressed soon after surgery with a new cerebellar nodule as well as spinal leptomeningeal disease which showed high-grade features on subsequent biopsy. Given the unusual clinical presentation, methylation profiling was performed. The tumor was matched to the class of an anaplastic pilocytic astrocytoma with a calibrated score of 0.4 and there was a chromosome 7 gain on the CNV plot. Given that this tumor sample sent for methylation profiling had very low tumor cellularity, this result was assessed as described by Capper et al. for intermediate-range calibrated scores with low tumor purity [12]. This result was thereby interpreted as suggesting that the tumor was more aligned with a malignant glial tumor than the initial diagnosis of a schwannoma. As the clinical presentation was also more consistent with that of a malignant glial tumor, this diagnosis was used to inform management decisions. While definitive treatment remains controversial for an aggressive tumor in this age range, the tumor board consensus was complete craniospinal radiotherapy.

#### Case 6

A 49-year-old male from an outside institution had a left frontal lesion that was subtotally resected. The histopathological differential diagnosis was PXA versus other high-grade glioma as it was not only superficial with compact growth and prominent eosinophilic granular bodies but also had frequent mitoses, microvascular proliferation, and necrotic areas. Methylation classification was pursued due to the differential diagnosis. The methylation class of the tumor was an IDH wildtype glioblastoma with a 0.99 calibrated score and the CNV plot showed EGFR amplification and PTEN loss. The integrated diagnosis was that of an IDH wildtype glioblastoma and the resulting significant change in management included concurrent chemoradiation and continued temozolomide.

#### Case 7

A 71-year-old female non-smoker presented with hemiparesis and an MRI showing a right frontal lesion having characteristics of a cystic primary glioma versus metastasis. After gross total resection, it was initially diagnosed as a TTF-1 positive metastatic high-grade neuroendocrine neoplasm with high cellularity, necrosis, minimal brain infiltration, and small cell phenotype. She received stereotactic radiosurgery postoperatively, fractionated radiotherapy plus cisplatin/etoposide for a 6-month recurrence, and surgery plus whole-brain radiotherapy for 12-month further progression. A primary tumor was not identified despite serial pan-imaging including chest CT scans, multiple negative biopsies (thyroid/breast nodule, lymph node, and uterine tissue), and laryngo-colonoscopies.

The case was flagged for methylation analysis as the course of disease progression was unusual with no primary systemic cancer being identified. The methylation classification and integrated diagnosis was an IDH wildtype glioblastoma (calibrated score of 0.94) with a characteristic gain of chromosome 7 and loss of chromosome 10 on the CNV plot. She then received temozolomide but progression continued and she is deceased 20 months from the initial surgery. Regarding the TTF-1 positivity, staining remained positive in the recurrent tumor and TTF-1 positivity has been reported rarely in glioblastomas [15].

## Discussion

DNA methylation profiling is an emerging powerful tool that can resolve diagnostic discrepancies and provide objective evidence to help classify challenging CNS tumor cases [11, 12, 16, 17]. There is increasing support within both the neuro-pathology and neuro-oncology communities for the increasing integration of molecular markers into diagnosis and treatment [6, 18]. Here, we show one of the first institutional experiences in rapidly adopting the methylation classifier and integrating it into practice to change the clinical management of brain tumor patients. We highlight that utilizing methylation testing in challenging CNS tumors not only refines diagnosis in 84% of the cases but also significantly impacts treatment decisions for a significant proportion (15%) of patients. Early recognition of the value of methylation testing at our institution resulted in rapid adoption by the multidisciplinary team managing CNS tumors. The necessary steps for adoption included early acquisition of technology, training to build medical expertise, and implementation of quality assurance measures.

Overall, of the 55 CNS tumor cases with challenging diagnoses, 84% had a clinically meaningful adjustment to the histopathological diagnosis with key components being a change in diagnosis for 24%, a resolved differential diagnosis for 31%, and the validation of new molecular markers to help with refinement of tumor classification for 29%. In the cohort of 1104 cases analyzed by the German Cancer Research Center that was initially used to validate the classifier, 12% received a change in diagnosis and 15% a molecular refinement [11]. In our series, we restricted the use of methylation profiling to challenging cases only and in this population, the utility and value of CNS tumor methylation classification were dramatically higher at 84%. This result supports the use of methylation profiling in those cases where there is uncertainty in diagnosis or where clinical suspicion of an alternative diagnosis is raised, in order to validate traditional histopathological diagnoses.

More importantly, we show that a significant subset of patients (15%) profiled experience impactful changes to their clinical management due to the additional diagnostic information. Unnecessary treatment and related side-effects were avoided in 6.4% of all patients and insufficient inappropriate treatment could be avoided in an additional 6.4%. The outlook on diagnosis was altered in another patient considering medically assisted death (MAID) based on the original diagnosis of an incurable glioblastoma. The decision to pursue MAID was reversed with the change in diagnosis after methylation profiling. Overall, avoiding unnecessary chemoradiation and associated risks/side effects, refining diagnosis to counsel the patients on outcome and overall quality of life, and choosing the best oncological treatment regimen are among the key points.

Furthermore, there is value to optimizing and streamlining molecular testing approaches and to ensure they are used in a judicious and cost-effective manner. Methylation classification and copy number plotting can be done with one array and can obtain results for IDH status, 1p/19q codeletion status, and MGMT methylation status, as the results between tests for these molecular features and the results by methylation profiling are highly concordant [19–24]. Optimal candidates for methylation profiling are those with a pathological diagnostic challenge or with limited tissue after a small biopsy precluding the application of multiple single tests. In diagnostically challenging cases a subset of these results may be obtained prior to pursuing methylation profiling but the cost of other tests can be avoided by profiling, including IDH1/2 staining and sequencing, FISH testing for 1p/19q codeletions in diffuse gliomas, and testing of MGMT methylation status in malignant gliomas. Avoiding unnecessary chemoradiotherapy treatment and unnecessary tests (i.e., tissue biopsies) to investigate uncertain diagnoses also significantly reduces care delivery costs. In our institution, methylation profiling costs approximately 550 USD per patient and, although the cost avoidance varies by case, this is substantially less than the expected cost of unnecessary tests and unnecessary chemoradiotherapy treatments. The role of methylation profiling is expected to expand as well, as new tumor subtypes by methylation signatures are identified such as the G-CIMP methylation pattern correlating with IDH mutation in glioma and its institutional role may expand [25].

A limitation of the methylation classifier is that it has an error rate of 1% and it is limited in distinguishing many grades II–III lesions, underlying the importance of the integrated diagnosis workflow that considers all pathological data [12]. The classifier determines MGMT status by a cutoff that maximizes the sum of the sensitivity and specificity, but there is emerging evidence of a more optimal clinically relevant cutoff that is significantly lower [14, 26]. Methylation turnaround times are variable depending on the institutional frequency of use but most are completed within 30 days and this time is decreasing with its expanding use. The median turnaround time of 25 days in our cohort is consistent with times seen by others [16].

## Conclusions

Overall, this work substantiates the value of DNA methylation profiling for diagnostically challenging CNS tumor cases by demonstrating that a substantial proportion receives changes to their diagnosis and treatment decisions after profiling. It is an example of how new technology can be rapidly integrated into practice and adopted clinically to improve patient outcomes. This technology avoids unnecessary treatment and testing related to misdiagnosis in challenging cases of CNS tumors in addition to reducing overall costs associated with individual molecular testing. Widespread adoption of genome-wide DNA methylation profiling as a diagnostic platform is important in order to keep delivery of care nimble and cutting-edge, allowing us to offer the best care to our patients.

## Data Availability

The datasets used and/or analyzed during the current study are available from the corresponding author on reasonable request.

## Abbreviations

CNS: Central nervous system
CNV: Copy number variant
DLGNT: Diffuse leptomeningeal glioneuronal tumor
DNET: Dysembryoplastic neuroepithelial tumor
FISH: Fluorescence in situ hybridization
FSRT: Fractionated stereotactic radiotherapy
FSRT: Single-dose stereotactic radiosurgery
GBM: Glioblastoma
IHC: Immunohistochemistry
MAID: Medically assisted death
NE: Neuroendocrine
NOS: Not otherwise specified
PXA: Pleomorphic xanthoastrocytoma
RT: Radiotherapy
RT: Temozolomide
SFT: Solitary fibrous tumor
UHN: University Health Network
WHO: World Health Organization
